# Potential Natural Dyes Food from the Powder of Prickly Pear Fruit Peels (*Opuntia* spp.) Growing in the Mediterranean Basin under Climate Stress

**DOI:** 10.1155/2020/7579430

**Published:** 2020-02-26

**Authors:** Mohammed Bourhia, Hamza Elmahdaoui, Samir Iben Moussa, Riaz Ullah, Ahmed Bari

**Affiliations:** ^1^Laboratory of Chemistry, Biochemistry, Nutrition, and Environment, Faculty of Medicine and Pharmacy, University Hassan II, Casablanca, Morocco; ^2^Laboratory of Food Technology and Quality, Regional Center for Agricultural Research in Marrakesh, National Institute for Agricultural Research, INRA, Marrakesh, Morocco; ^3^Medicinal Aromatic and Poisonous Plants Research Center, College of Pharmacy, King Saud University, P.O. Box 2457, Riyadh 11451, Saudi Arabia; ^4^Central Laboratory, College of Pharmacy, King Saud University, P.O. Box 2457, Riyadh 11451, Saudi Arabia

## Abstract

**Background:**

Barbary fig (*Opuntia* spp), called prickly pear, is a cultivated species belonging to family Cactaceae. It produces fruits one time per year during a short season. It is largely spreading in the Mediterranean countries. The aim of the study was to investigate the physicochemical and biochemical analysis of powder of prickly pear fruit peels of three Moroccan varieties (Aakria, Derbana, and Mles). *Material and Methods*. Both physicochemical analysis (humidity, water activity, Brix, ash content, pH, and total titratable acidity) and biochemical analysis (total carotenoid content, betalain content, total polyphenolic content, and ascorbic acid content) were effectuated according to protocols reported within the present research work.

**Results:**

Regarding the physiochemical analysis, the humidity of powder of prickly pear fruit peels ranged from 10.173 ± 0.002 to 15.27 ± 0.02%. The water activity (aw) ranged from 0.17 ± 0.002 to 0.336 ± 0.002. The values of Brix ranged from 68.67 ± 0.33 to 74.22 ± 0.51° Bx. pH values ranged from 5.41 ± 0.01 to 5.62 ± 0.01. The total titratable acidity values ranged from 0.644 ± 0.014 to 0.76 ± 0.007 g citric acid/100 g DM (dry matter). The ash content ranged from 1.02 ± 0.02 to 11.41 ± 0.03 g/100 g DM. Regarding the biochemical analysis, the total carotenoid content ranged from 5.14 ± 0.10 to 9.79 ± 0.06 mg/g of FM (Fresh matter). The total betalain content ranged from 2 ± 0.69 to 37.66 ± 2.65 mg/100 g of DM. The total polyphenolic content ranged from 1739.92 ± 13.69 to 2409.66 ± 43.65 mg gallic acid equivalent (GAE)/100 g of DM. The ascorbic content values ranged from 186.78 ± 13.23 to 294.04 ± 5.81 mg/100 g of DM.

**Conclusion:**

The results showed an interesting richness of the investigated powder in pigments (betacyanins, indicaxanthins, and carotenoids) and nutritional compounds including sugar, proteins, and vitamins that make this powder interesting for being used as a dye in food.

## 1. Introduction

Humans have always used plants as a source of food. From the earliest times, plants constitute a food reservoir of both animals and humans. Plants have been largely used in medicine to cure disease and provide drugs. Plants give people on earth pleasure through gardening [1]. People on the earth still linked to plants for food whether directly or as feed for animals breeding. Agriculture plays a key role in food crop production and the civilizations of the world. Nowadays about 7,000 species of plants are used for food though most of today's only 30 species are used intensively for food. Nonfood products such as essential oils, natural dyes, and pigments are also derived from plants [2].

Prickly pear (*Opuntia* spp.) called “Barbary fig” is one of the most important perennial crop species, which has the ability to adapt to stress even at low soil potential. It is becoming more important for social and economic development. Therefore, Barbary fig has drawn attention for being valorized and listed as promising projects for the development of rural areas. The prickly pear is a plant native to Mexico areas. It was introduced in the 16th century to the Mediterranean basin such as North of Africa by Spaniards, where it was naturalized [3, 4]. Nowadays prickly pear is found in a range of climates ranging from the Mediterranean to tropical [5]. Barbary fig has widely grown in Moroccan rural landscape. Its geographical distribution is quite extensive; it is extending from the coastal regions of Sidi Ifni to Tangier city. In the Tiznit land, the crops of Barbary fig have occupied a total area of 17,600 ha. 65% of which are in the region of Sidi Ifni and Aït Baâmrane. In the study area, more than 1500 known species of prickly pear are springing from genera *Opuntia* [6].

Barbary fig fruit possesses interesting chemical compounds, which can provide it with good nutritional value. High genetic variability is preserved in the Moroccan prickly pear. Several varieties have been distinguished by the flowering period (early or late), the color of the flower, the color of fruit and pulp, the shape of fruits (ovoid, round and oblong), the organoleptic criteria of fruits, and the antioxidant power [7]. The fruits of the prickly pears containing betalain pigments that provide them with good potential for use as natural dyes or cosmetics. These fruits contain betacyanins, in addition to betaxanthins [8, 9].

The present study aims to valorize the powder of dried peels of three prickly pear varieties (Aakria variety, Derbana variety, and Mles variety) growing in dry Moroccan zones in terms of quality and stability with different humidity levels (15% and 10%).

## 2. Material and Methods

### 2.1. Study Area

**Figure 1:**
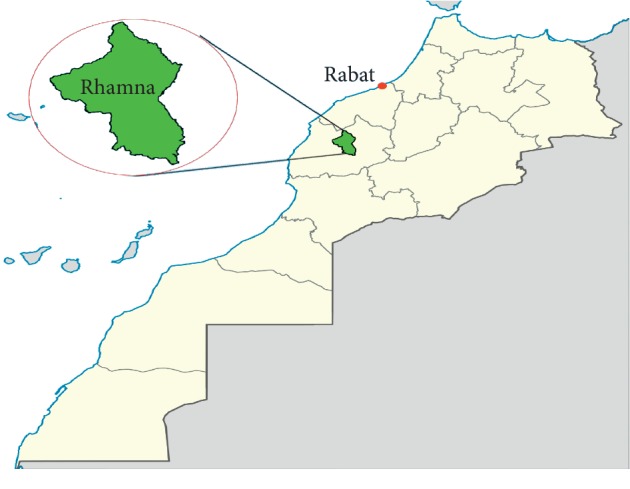
Geographical position of the study area (region of Rhamna, Morocco).

The investigated varieties of Barbary fig were collected from Rhamna land, an area located at the central west of Maghreb (Morocco), about 100 km Northwest of Marrakech touristic city (Figure 1).

#### 2.1.1. Regional Climatic Conditions

**Figure 2:**
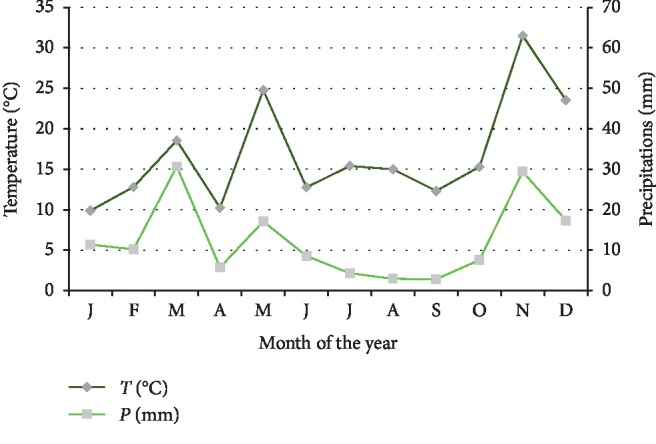
Diagram of Gaussen and Bagnouls of the study area (2015-2016).

Rhamna land is described by arid Mediterranean climates over the year (cold winter and dry summer) according to earlier reported data (Emberger's Pluviothermic Quotient: *Q*^2^ = 20.6). Insufficient and irregular annual rainfall extends throughout the study area (Xeric Climate). The annual mean of precipitation is about 130 mm (methodological station of Ben Guerir, 2017). According to Gaussen and Bagnouls diagram, Rhamna land classified as dry area (Figure 2).

### 2.2. Plant Material

**Figure 3:**
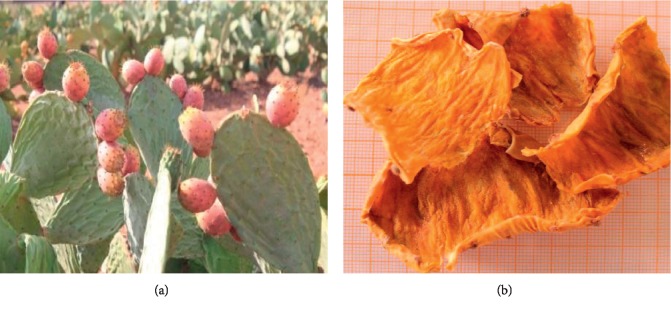
Aakria variety (*Opuntia ficus-indica*): (a) Aakria Plant. (b) Aakria Peels.

**Figure 4:**
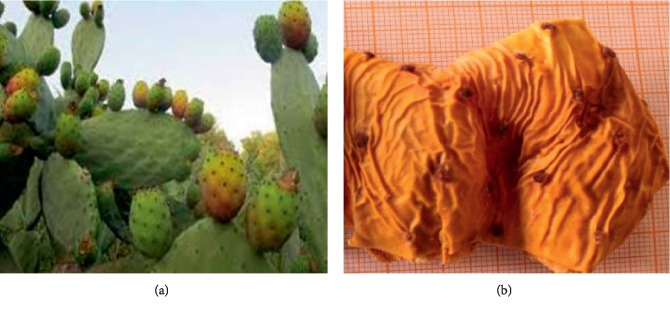
Derbana variety (*Opuntia megacantha*): (a) Derbana Plant. (b) Aakria peels.

**Figure 5:**
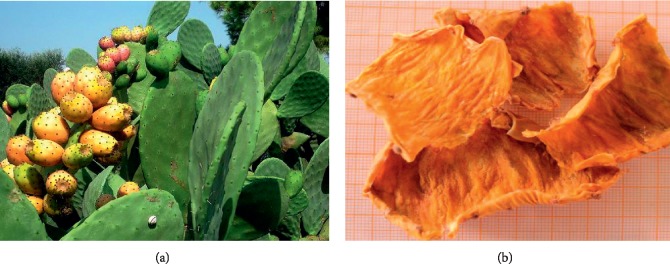
Mles variety (*Opuntia ficus-indica*): (a) Mles Plant. (b) Mles Peels.

The investigated varieties of Barbary fig were collected from Rhamna land at the end of maturity in August of 2015. The studied species were identified by a botanist. The fruits were peeled in order to obtain the requested peels; the fresh peels were left at room temperature for the drying process. The dried peels were ground into powder. The studied varieties are presented in Figures 3–5.

#### 2.2.1. Preparation of Powder of Prickly Pear Fruit Peels

Two types of powders were prepared from the fruit peels of the investigated varieties in order to evaluate the stability and the quality of the present product:

- A powder at moisture content 15%
- A powder at moisture content 10%

The choice of these moisture levels was not randomly but was based on previous work, in which it was reported that the peels of *Opuntia* spp. got dried up at moisture levels ranged from 10% to 18% [7, 9].

The peels were placed into an oven at 60°C for drying. To reach the fixed moisture levels (15% and 10%), preliminary tests were carried out by drying the peels and determining the reduction of humidity content as a function of time of drying. Once the desired humidity level was reached, the drying was stopped and the drying time was determined. The dried peels were then crushed and stored in hermetically sealed glass jars away from light.

### 2.3. Physicochemical Analysis of Powder of Prickly Pear Fruit Peels

#### 2.3.1. Humidity Determination

5 g of powder was placed in a vacuum oven at 105°C for 48 hours. At the end of the drying, the obtained final weight of the sample was measured again. Three repetitions were performed according to a previously reported method [10].

The humidity % was determined as follows:

(1) $$\begin{array}{c} \text{Humidity }\%=\left(\frac{\text{Iw}-\text{Fw}}{\text{Iw}}\right)*100, \end{array}$$

where Iw is the weight of the test portion measured before steaming (g) and Fw is the weight of the test portion measured after steaming (g).

#### 2.3.2. Water Activity Determination

5 g of powder was placed in an aw-meter at a temperature of 25°C for 2 hours deemed sufficient to reach the balance between the sample and the surrounding atmosphere. Then aw was read using aw-meter. Three repetitions were carried out.

#### 2.3.3. pH Determination

The pH of powder of prickly pear fruit peels was determined using a pH meter. A solution was prepared with distilled water then neutralized at a ratio of 1 : 6 (the studied powder: volume of distilled water).

#### 2.3.4. Determination of Titratable Acidity

This parameter was determined as follows: 25 g of powder supplemented with distilled water and boiled for 30 min. The obtained solution was filtered, then titrated with NaOH (0.1 N) until pH = 8.1.

The results were reported in *μ*g citric acid equivalent per g of DM:

(2) $$\begin{array}{c} \text{Total titratable acidity}\left(\text{g c.a}/100\text{ g}\right)=\frac{\left(\left(\text{VNaOH added }*\text{ NNaOH }*\;0.07\right)/\text{Vs}\right)}{100}*\text{Sw,} \end{array}$$

where VNaOH is the NaOH volume (in ml); NNaOH is the NaOH normality; Vs is the sample of titrated volume (in ml); c.a: citric acid; 0.07 is the coefficient of citric acid; and Sw is the weight of sample.

#### 2.3.5. Brix Determination

Regarding the analysis of dried peels, the test portion was 1 g solubilized in 10 ml of distilled water at a ratio of 1 : 10 (used powder: used distilled water). The assay was performed in triplicate. The results were reported in degree Brix (Bx).

#### 2.3.6. Determination of Ash Content

2 g of powder of prickly pear fruit peels was placed for incineration at a temperature of 500°C for 7 hours; three repetitions were carried out. The ash content was reported as a percentage relative to dry matter (DM):

(3) $$\begin{array}{c} \text{Ashes in }\text{g}/100\text{ g}\text{DM}=\left[\frac{\left(\text{Iw}-\text{Fw}\right)*100}{100-H}\right]*100, \end{array}$$

where Iw is the measured weight of the test sample before incineration (g); Fw is the measured weight of the test sample after incineration (g); and *H* is the humidity (%).

#### 2.3.7. Colour Determination

In the current work, the color was determined for powders at 15% and 10% of humidity content. A colorimeter was used for measuring the parameters of the color or space Lab (*L*^*∗*^, *a*^*∗*^ and *b*^*∗*^). *L*^*∗*^ value ranged from 0 (black) to 100 (white), *a*^*∗*^ value ranged from −100 (green) to +100 (red), and the value of *b*^*∗*^ ranged from −100 (blue) to +100 (yellow). When the values of *L*^*∗*^, *a*^*∗*^ and *b*^*∗*^ increase, the color is more chromatic, by the time the value of neutral colors such as white, gray, and black tends to be zero.

Chroma was calculated as follows:

(4) $$\begin{array}{c} C={\left[a^{*2}+b^{*2}\right]}^{0.5}. \end{array}$$

where *C*^*∗*^ is the chroma (represents the purity of the color); *a*^*∗*^ represents a range of 200 levels on the red axis (+100) ⟶ green (−100) through gray (0); and *b*^*∗*^ represents a range of 200 levels on the yellow axis (+100) ⟶ blue (−100) through gray (0).

### 2.4. Biochemical Analysis of Powder of Prickly Pear Fruit Peels

#### 2.4.1. Total Carotenoid Content Determination

1 g of powder of prickly pear fruit peels was mixed with 10 ml of solvents (50% hexane, 25% acetone, and 25% ethanol). The mixture was vortexed for 1 min and centrifuged at 6500 g for 5 min at a temperature of 5°C. The supernatant containing the pigments was recovered again then adjusted with hexane. OD (Optic density) was read at 450 nm according to earlier described methods [11]. The assay was performed in triplicate:

(5) $$\begin{array}{c} \text{Total carotenoid content}\left(\mu \text{g eq }\beta -\text{carotene}/\text{g}\right)=\frac{A_{450}*V*106}{2500*100*\text{Sw}}, \end{array}$$

where *A*_450_ is the read absorbance at 450 nm; *V* is the analysed volume of the sample (1 ml); and SW is the weight of Sample (g).

#### 2.4.2. Betalain Content Determination

0.5 g of powder of prickly pear fruit peels was mixed with methanol 80% (v/v) for one minute and centrifuged at 4000 rpm for 20 minutes. The recovered supernatant was read by spectrophotometer at 480 nm and 538 nm for the determination of both, betaxanthins and betacyanins, respectively, according to earlier data [11]. Betalain content was determined as follows:

(6) $$\begin{array}{c} \text{Betalain content}\left(\mu \text{g}/100\text{ g}\right)=\left(\frac{\left(A*\text{Df}*\text{Mw}*1000/\left(\xi *\text{Wt}\right)\right)}{\text{Sw}*100}\right), \end{array}$$

where *A* is the absorbance; Df is the dilution factor; Wt is the width of the tank (1 cm); *ξ* is the molar extinction coefficient (L/mol *∗* cm); Mw is the molecular weight (g/mol); *λ* is the wavelength (nm); and Sw is the sample weight.

#### 2.4.3. Total Polyphenol and Ascorbic Acid Content Determination

*(1) Extraction*. Powder of Barbary fig fruit peels was placed for extraction with 10 ml of acetone (70%) and (80%). The mixture was centrifuged at 5000 rpm for 30 min. The extract obtained was used for dosing the total polyphenol and ascorbic acid content.

#### 2.4.4. Total Polyphenols Determination

Briefly, 25 *μ*l of powder of prickly pear peels extract was added to 0.475 ml of methanol and 2.5 ml of Folin-Ciocalteu reagent (10%). After 2 minutes, 2 ml of sodium carbonate (at 75 g/l) was added. The mixture was placed under incubation at 50°C for 15 minutes and then cooled rapidly. The absorbance was read at 760 nm.

The findings were reported in mg gallic acid equivalent (EAG)/100 g of DM through a standard curve. The assay was performed in triplicate.

#### 2.4.5. Ascorbic Acid Content

500 *μ*l of extract prepared from powder of prickly pear fruit peels was added to 3.5 ml of distilled water then mixed. 2 ml of the current mixture was deposited on an OASIS cartridge embedded with 3 ml of MeOH + 2 ∗ 3 ml of distilled water. 0.5 ml of the obtained solution was measured. The findings were reported in mg/100 g of DM. The current assay was repeated in triplicate.

### 2.5. Statistical Analysis

The results were reported as the average ± standard deviation. Analysis of variance was performed using one-way ANOVA. For performing multiple comparisons between means, the Student–Newman–Keuls method was adopted. A significant difference was taking into account at *p* less than 0.05.

## 3. Results and Discussion

### 3.1. Physicochemical Analysis of Powder of Prickly Pear Fruit Peels

#### 3.1.1. Colour of Powder of Dried Peels

Colour is a quality index that plays an important role in the commercial value of food products. Variation of color is closely related to the progression of fruit ripening and other factors that depend on technological treatments [12].

**Figure 6:**
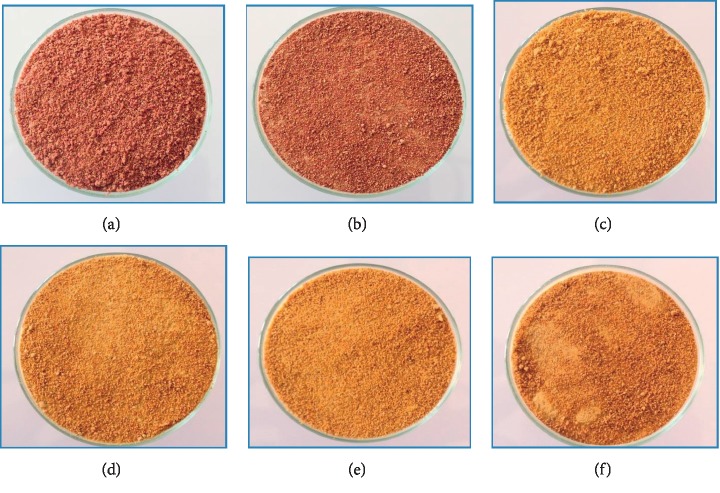
Powders with 15% and 10% moisture content of prickly pear fruit peels (Aakria variety, Derbana variety, and Mles variety). (a) Akaria powder with 15% moisture content. (b) Aakria powder with 10% moisture content. (c) Derbana powder with 15% moisture content. (d) Derbana powder with 10% moisture content. (e) Mles Powder with 15% moisture content. (f) Mles powder with 10% moisture content.

Powder color of the studied peels was red-violet for Aakria variety and yellow-orange for Derbana and Mles varieties (Figure 6). The color parameters (*L*^*∗*^, *a*^*∗*^, *b*^*∗*^ and chroma *C*^*∗*^) of powders of the current varieties with two moisture contents 15 and 10% are presented in Table 1.

Analysis of variance showed that the clarity (*L*^*∗*^) differed significantly between powders of Aakria and Derbana varieties regarding the two moisture levels (15 and 10%) (*p* < 0.01). The luminosity of color decreased due to peels drying (Table 1). The reduction in luminosity was reported in the order of 12.7%, 7.9% and 1.8% for Aakria, Derbana, and Mles respectively.

The values *a*^*∗*^ represent that the range of color green/red was positive that indicates an appearance of red color. These were very high in the powder of Aakria variety compared to other varieties (Derbana and Mles). These results could explain the richness of this variety in red pigments of betacyanin. The monitoring of the current parameter evolution as a function of peels drying showed a significant decrease in the order of 8.29%, 3.49%, and 5.76%, respectively, concerning Aakria variety, Derbana variety, and Mles (*p* < 0.05). The highest decrease was recorded in peels of Aakria; this could be explained by the degradation of betalain under drying effect.

The values *b*^*∗*^ represent that the range of yellow/blue color was positive regarding the studied powder that indicates an appearance of yellow color. When moving from a humidity level of 15 to 10%, a significant decrease of 0.57%, 2.13%, and 5.95% was registered in powders of peels prepared from Aakria, Derbana, and Mles, respectively (*p* < 0.05). This decrease could be due to the degradation of yellow pigments (indicaxanthins) under the drying effect.

The decrease of parameters values *a*^*∗*^ and *b*^*∗*^ did not induce a significant reduction of chroma *C*^*∗*^ values of the studied powders at 15 and 10% humidity level. The values of chroma color 40.84 ± 1.12 and 40.89 ± 1.45 obtained for yellow-orange varieties (Derbana and Mles, respectively) were higher than those reported in earlier data regarding fresh peels of prickly pear growing in the South of Morocco (19.12 and 18.27) [13]. The remarkable difference could be due to the richness of the studied peels in pigments (betalains and carotenoids). The present results were in accordance with previous data report on color of dried peels of another variety of Barbary fig fruit 27.56 [14].

The current results showed that the reduction of moisture content from 15 to 10% influenced the color of fruit peels by decreasing of parameter values of *a*^*∗*^ and *b*^*∗*^. This decrease was resulted due to drying of peels and could be explained by involving of browning reactions.

#### 3.1.2. Humidity

The values of humidity content of dried peels at two levels 10% and 15% ranged from 10.173 ± 0.002 to 15.27 ± 0.02%, respectively (Table 2). Concerning humidity content, the powder of all studied varieties at 10% did not show a significant difference (*p* > 0.05). However, there was a significant difference between varieties powders at 15% (*p* < 0.01).

#### 3.1.3. Water Activity

ANOVA analysis showed a significant difference in water activity between powders of the investigated varieties at 15% and 10% moisture content (*p* < 0.001). The lowest value of water activity was recorded in the powder of peels at 10% moisture content meanwhile the highest value was registered in powder at 15% humidity (Table 2). The current values of aw that ranged from 0.173 ± 0.002 to 0.336 ± 0.002 were found lower than those reported in previous literature regarding another variety of Berbay fig, El Beida 0.410 [15]. The low aw values reported in our work can ensure chemical and microbial stability by inhibiting the growth of microorganisms. Therefore the found value increases the aptitude of powder conservation.

#### 3.1.4. Brix

The Brix values of Derbana variety powder at 15% moisture content and Mles variety powder at 10% ranged from 68.67 ± 0.33 to 74.22 ± 0.51°Bx, respectively. A significant difference was recorded between the powders of studied varieties at two moisture levels (*p* < 0.05). However, no significant difference was recorded between the powders of Aakria and Mles varieties at 15% humidity (Table 2). In the current research, Brix values of powders at 15% moisture content were lowest than these recorded in powders at 10% due to the concentration of soluble dry matter in powder with low humidity.

The values of Brix found in the investigated powders of dried peels were higher than those measured in peels of oranges 46.241 ± 0.015°Bx [16].

#### 3.1.5. pH

pH values of Aakria and Derbana varieties ranged from 5.41 ± 0.01 to 5.62 ± 0.01, respectively. ANOVA analysis showed a significant difference between the pH of dried peels at 15 and 10% humidity content of both Aakria and Derbana varieties (Table 2). Lower values were reported in earlier data 4.5 ± 0.1 and 4.4 ± 0.1 regarding fresh and dried *Opuntia ficus-indica* peels, respectively [17].

#### 3.1.6. Titratable Acidity

ANOVA analysis revealed a significant difference between powders of both, Derbana and Mles varieties at two moisture levels (15 and 10%) (*p* < 0.05) (Table 2). The lowest total titratable acidity value 0.644 ± 0.014 g citric acid/100 g DM and the highest 0.763 ± 0.007 g citric acid/100 g of DM were attributed to powders of Aakria variety at 10% moisture content and Derbana variety at 15% moisture content, respectively. The values of the total titratable acidity obtained in the current work were close to previous found data 0.60 ± 0.10 g citric acid/100 g DM [17].

#### 3.1.7. Ash Content

Concerning all varieties, ANOVA analysis revealed no significant difference between the ash content of studied powders at two moisture levels (15 and 10%) (Table 2). The obtained values of ash content ranged from 11.02 ± 0.02 to 11.41 ± 0.03 g/100 g DM. These values were in agreement with earlier found data about ash content of *Opuntia ficus-indica* 11.5 g/100 g DM [16]. The current findings showed that the powders of prickly pear peels possess high levels of mineral matter. On the other hand, the drying of fresh peels did not affect negatively the mineral content. As reported in previous literature, the prickly pear fruit peels can be considered as promising sources of magnesium, calcium, sodium, and potassium [17].

### 3.2. Biochemical Analysis of Powder of Prickly Pear Fruit Peels (Aakria, Derbana, and Mles)

#### 3.2.1. Pigment Content

**Figure 7:**
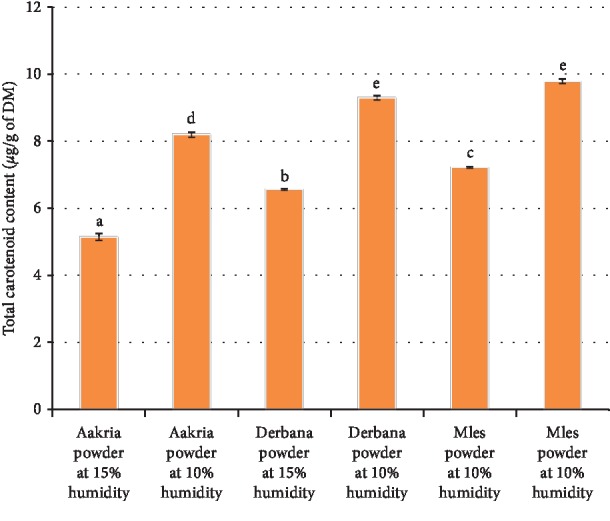
Total carotenoid content of powder of prickly pear fruit peels (Aakria variety, Derbana variety, and Mles variety). Means with the same letter do not present a significant difference at *p* < 0.05.

*(1) Total Carotenoid*. Regarding all investigated varieties in this study, the total carotenoid content in powders ranged from 5.14 ± 0.10 to 9.79 ± 0.06 mg/100 g of DM (Figure 7). ANOVA analysis revealed a high significant difference between the powders at the two moisture levels (15 and 10%) (*p* < 0.001)

The values of total carotenoid reported in the present research work were lower than those previously reported regarding dried peels of *Opuntia ficus-indica* at 18.5% humidity content 12.76 ± 0.20 mg/100 g of DM [17].

We reported that the total carotenoid contained in powders increased for all studied varieties when moving from 15% to 10% moisture content. Therefore, the degree of drying influences by the carotenoid content in the studied powders. This increase could be explained by the concentration of carotenoids in powder at low humidity.

**Figure 8:**
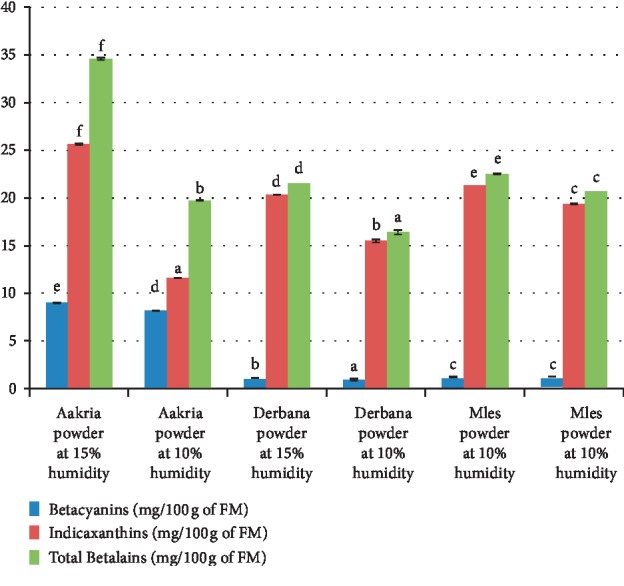
Betalain content (betacyanins, indicaxanthin, and total betalains) of powder of prickly pear fruit peels (Aakria variety, Derbana variety, and Mles variety). Means with the same letter do not present a significant difference at *p* < 0.05.

*(2) Betalains*. Betalains values found in the studied powders of all studied varieties ranged from 2 ± 0.69 to 37.66 ± 2.65 mg/100 g of DM. ANOVA analysis showed a highly significant difference between the total betalain content of powders of studied varieties peels regarding both moisture levels 15 and 10% (*p* < 0.001) (Figure 8).

Dried peels powder of Aakria variety possessed a higher level of betacyanins (red pigments) compared to powders of Derbana and Mles varieties. Meanwhile, the powder of Derbana was richer in indicaxanthins (yellow pigments) than Aakria variety. The values of betalain content registered for powders of Derbana and Mles varieties were similar to those found in peels of yellow-orange [18]. The powder of *Opuntia megacantha* (Derbana) possesses a higher level in indicaxanthins than betacyanins. The betacyanin content of Aakria variety was close to that found in previous literature concerning dried peels of *Opuntia ficus-indica* [14].

### 3.3. Phenolic Content

**Figure 9:**
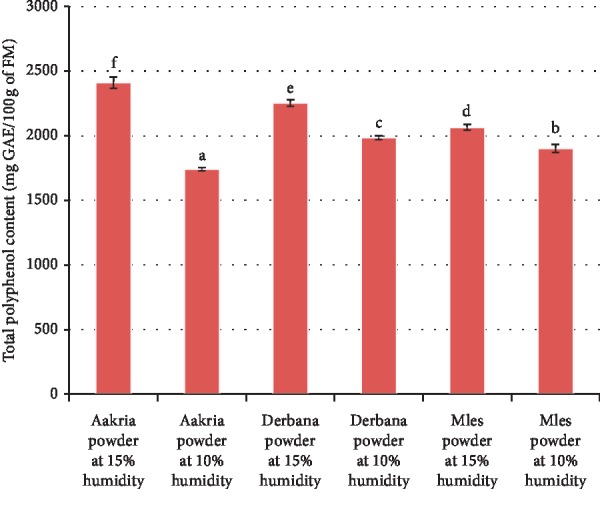
Phenolic content of powder of prickly pear fruit peels (Aakria variety, Derbana variety, and Mles variety). Means with different letters present a significant difference at *p* < 0.05.

Regarding phenolic content, there was a highly significant difference between the powders of the three studied varieties at the two moisture levels (15 and 10%) (Figure 9).

The total polyphenols values found in the studied powders ranged from 1739.92 ± 13.69 to 2409.66 ± 43.65 mg gallic acid equivalent (GAE)/100 g DM. These results were in accordance with published data about total polyphenols of orange dried peels (1968 ± 2 mg GAE/100 g of DM) [16]. We noted that drying induced a significant reduction in the order of 28%, 12%, and 8% in total polyphenols of powders of Aakria, Derbana, and Mles respectively. The total polyphenols of Aakria variety were the least stable with increasing drying from 15 to 10% humidity.

The observed decrease in total polyphenols of the investigated powders due to drying from 15 to 10% humidity could be explained by the degradation phenomena of these compounds under heating. Our findings were completely in agreement with earlier works [19], in which it was reported that the degradation of polyphenols induced by heat treatment between 50 and 60°C.

### 3.4. Ascorbic Acid Content

**Figure 10:**
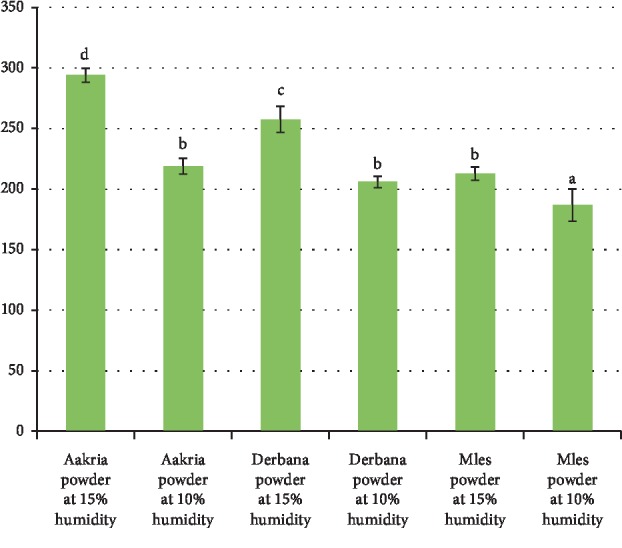
Ascorbic acid content of powder of prickly pear fruit peels (Aakria variety, Derbana variety, and Mles variety). Means with the same letter do not present a significant difference at *p* < 0.05.

Regarding all prickly pear varieties, there was a significant difference between ascorbic acid contained in powders at the two moisture levels, 15 and 10% (*p* < 0.001) (Figure 10).

Aakria powder at 15% humidity and Mles powder at 10% humidity showed ascorbic acid values 186.78 ± 13.23 and 294.04 ± 5.81 mg/100 g of DM, respectively. These obtained findings were in consent with previous data [17], in which it was reported that the ascorbic acid content revealed in dried peels of *Opuntia ficus-indica* was at about 288.67 ± 4.16 mg/100 g of DM. The highest values of ascorbic acid content were recorded in dried peels at 15% humidity compared to those dried at 10% humidity. The remarkable reductions in powders ascorbic acid content by drying from 15% to 10% moisture were in the order of 25%, 20%, and 12% for all varieties Aakria, Derbana, and Mles, respectively.

We can confirm that drying influences the ascorbic acid content in the dried peels. Heat treatment induces the degradation of ascorbic and affects negatively other quality parameters [20, 21].

## 4. Conclusion

The present research work gives interesting data about physiochemical and biochemical analysis of powder of prickly pear fruit peels. Through the reported results we can affirm that the dried peels of prickly pear fruit (powders) could constitute a promising source of natural dyes which may be exploited in food and cosmetic because of their richness in pigments (betacyanins, indicaxanthins, and carotenoids). The powder of prickly pear fruit peels also possesses a nutritional composition including sugar proteins, vitamins that make this powder interesting for human food.

## Figures and Tables

**Table 1:** Parameters of powder color of prickly pear fruit dried peels (Aakria, Derbana, and Mles) at moisture levels 15% and 10%.

Parameters of powder color	Aakria		Derbana		Mles	
	Powder at 15% humidity content	Powder at 10% humidity content	Powder at 15% humidity content	Powder at 10% humidity content	Powder at 15% humidity content	Powder at 10% humidity content
*L* ^*∗*^	43.14 ± 5.22^b^	37.93 ± 2.92^a^	56.57 ± 2.26^d^	51.79 ± 1.97^c^	53.70 ± 2.02^c^	52.08 ± 3.11^c^
*a* ^*∗*^	19.53 ± 1.36^d^	17.91 ± 1.05^c^	11.17 ± 0.69^ab^	10.78 ± 0.73^a^	11.28 ± 0.17^b^	10.63 ± 1.04^a^
*b* ^*∗*^	19.43 ± 1.74^b^	19.32 ± 2.53^a^	39.38 ± 1.13^d^	38.54 ± 1.50^cd^	39.47 ± 1.33^d^	37.26 ± 1.50^c^
Chroma (*C*^*∗*^)	27.93 ± 0.70^a^	27.56 ± 1.68^a^	40.84 ± 1.12^b^	40.13 ± 1.44^b^	40.89 ± 1.45^b^	39.43 ± 0.94^b^

*L* ^*∗*^ represents the luminosity (*L* = 0 for black, 100 for white); *a*^*∗*^ represents red/green color values; *b*^*∗*^ represents yellow/blue color values; Colour index: Chroma *C*^*∗*^ = [a ∗ 2 + b ∗ 2]^0.5^. The results were reported as average average ± standard deviation. The reported values with the same letter in the same line did not differ significantly at *p* < 0.05.

**Table 2:** Physicochemical analysis of powders of prickly pear fruit peels at moisture levels 15 and 10% (Aakria, Derbana, and Mles).

Criteria	Plants		Derbana		Mles	
	Powder at 15% humidity content	Powder at 10% humidity content	Powder at 15% humidity content	Powder at 10% humidity content	Powder at 10% humidity content	Powder at 15% humidity content
Humidity (%)	15.27 ± 0.02^c^	10.80 ± 0.05^a^	15.57 ± 0.02^d^	10.71 ± 0.01^a^	15.04 ± 0.07^b^	10.71 ± 0.01^a^
Water activity	0.30 ± 0.001^d^	0.187 ± 0.008^b^	0.33 ± 0.002^e^	0.176 ± 0.001^a^	0.28 ± 0.001^c^	0.17 ± 0.002^a^
Brix (°Bx)	70.67 ± 0.67^b^	73.50 ± 0.33^d^	68.67 ± 0.33^a^	72.67 ± 0.47^c^	71.22 ± 0.19^b^	74.22 ± 0.51^e^
pH	5.62 ± 0.01^c^	5.52 ± 0.07^b^	5.48 ± 0.01^b^	5.41 ± 0.01^a^	5.54 ± 0.01^b^	5.54 ± 0.01^b^
Total titratable acidity (g citric acid/100 g of FM)	0.64 ± 0.014^a^	0.637 ± 0.044^a^	0.763 ± 0.007^c^	0.693 ± 0.010^b^	0.726 ± 0.011^b^	0.639 ± 0.008^a^
Ash content (g/100 g of DM)	11.41 ± 0.03^a^	11.37 ± 0.06^a^	11.20 ± 0.01^a^	11.24 ± 0.01^a^	11.02 ± 0.03^a^	11.02 ± 0.02^a^

The results were reported as average ± standard deviation. Values with the same letter in the same line did not differ significantly at *p* < 0.05.

## Data Availability

The data used to support the findings of this study are included within the article.
